# Uterine Arteriovenous Malformation: Diagnostic and Therapeutic Challenges

**DOI:** 10.3390/diagnostics14111084

**Published:** 2024-05-23

**Authors:** Luisa Clavero Bertomeu, Laura Castro Portillo, Cristina Fernández-Conde de Paz

**Affiliations:** Department of Obstetrics and Gynecology, Valme University Hospital, 41014 Sevilla, Spain; luisa@dclavero.es (L.C.B.); lauracastroportillo@gmail.com (L.C.P.)

**Keywords:** uterine arteriovenous malformation, myometrial hypervascularisation, peak systolic velocity, Doppler ultrasound

## Abstract

Uterine arteriovenous malformations are a rare cause of puerperal haemorrhage, but their incidence is increasing due to both improved diagnosis and the more frequent use of uterine surgery in recent years. The use of ultrasound, both B-mode and Doppler, is recommended for diagnosis and follow-up, as it has been shown to be the simplest and most cost-effective method. Endometrial thickening associated with an anechoic and vascular intramiometrial structure is very useful for diagnosis and can help to exclude other causes of dysfunctional bleeding. Pulsed Doppler shows low-resistance vessels and high pulsatility indices with a high peak systolic velocity (PSV). In a healthy myometrium, the vessels have a peak systolic velocity of 9–40 cm/s and a resistance index between 0.6 and 0.8, whereas in the case of AVMs, the systolic and diastolic velocities are 4–6 times higher (PSV 25–110 cm/s with a mean of 60 cm/s and a resistance index of 0.27–0.75 with a mean of 0.41). For treatment, we must individualise each case, taking into account haemodynamic stability, the patient’s reproductive wishes, and the severity of the AVM as assessed by its size and PSV.

## 1. Introduction

Uterine arteriovenous malformations (AVMs) are an uncommon cause of puerperal metrorrhagia and haemorrhage [1]. Understanding them is important as they are potentially fatal [2,3] and because there has been an increase in their occurrence in recent years due to the new diagnostic techniques available.

Their clinical and radiological suspicion is fundamental since common interventions to control uterine haemorrhage, such as curettage in the case of a uterine arteriovenous malformation, can lead to massive haemorrhage and a fatal outcome [1].

Uterine arteriovenous malformations can be congenital or acquired, the latter being the most frequent [1]. Congenital ones originate from an alteration in the embryological differentiation of primitive vascular structures, creating abnormal vascular connections (fine capillaries intertwined with myometrial vessels) that can reach large pelvic vessels in addition to the uterine arteries (especially in women of reproductive age) [1,4].

Acquired arteriovenous malformations constitute true arteriovenous fistulas between the intramural arterial branches and the myometrial venous plexus. They are related to iatrogenic uterine trauma, such as obstetric curettage, uterine surgery (caesarean section, myomectomy), or even traumatic childbirth [5,6]. Other less frequent causes of AVMs are those coexisting with endometrial cancer, gestational trophoblastic disease, or infections [1,7].

Abnormal intramiometrial and endometrial vessel formation can cause severe uterine bleeding, constituting a threat to life and hindering future pregnancies. We describe the case of a 40-year-old patient diagnosed with a uterine AVM after severe metrorrhagia secondary to curettage for delayed abortion.

The main aim of our study was to review the diagnostic and therapeutic management of uterine arteriovenous malformation, a rare but potentially fatal entity.

## 2. Case Report

A 40-year-old woman presented to the gynaecological emergency department with severe metrorrhagia two months after aspiration curettage for delayed abortion (obstetric formula G1A1). She had no allergies or other medical or surgical history of interest. Upon arrival at the emergency department, moderate bleeding was observed.

**Figure 1:**
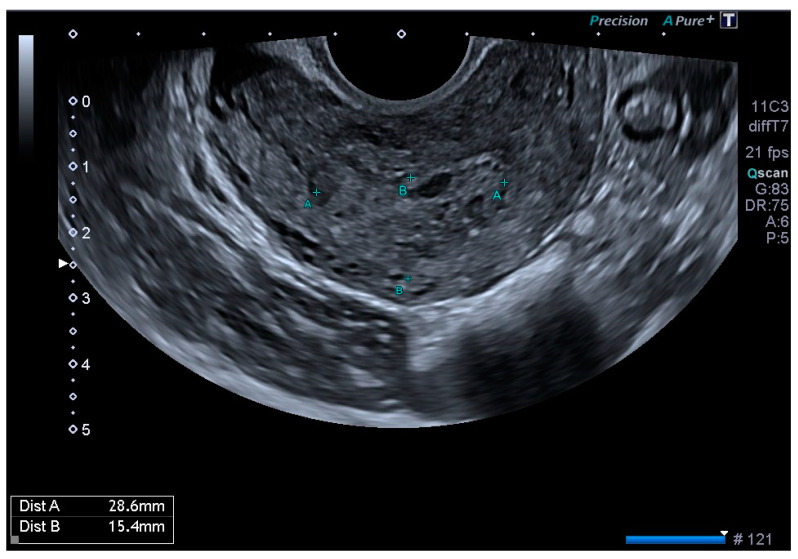
Transvaginal ultrasound with suspected uterine arteriovenous malformation.

**Figure 2:**
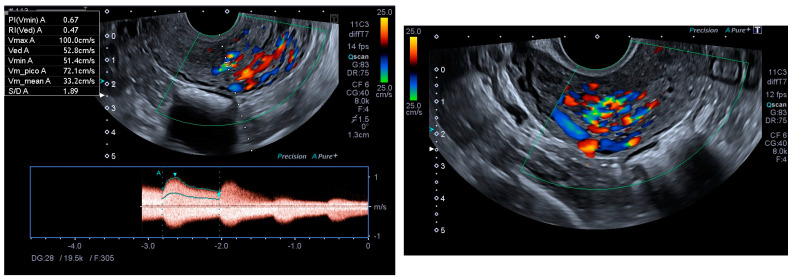
Colour and pulsed Doppler studies of the uterine arteriovenous malformation.

Ultrasonography showed an endometrial cavity occupied by a protruding formation in the uterine cavity measuring 38 × 15 mm, compatible with a uterine AVM (Figure 1). The presence of vascularisation of the right lateral uterine wall was noted, and pulsed Doppler showed a peak systolic velocity > 100 cm/s (Figure 2).

Following these findings, the patient was advised to avoid pregnancy, so combined hormonal contraception was prescribed, and an angio-CT scan of the pelvis was requested with a referral to the Vascular Surgery Department.

**Figure 3:**
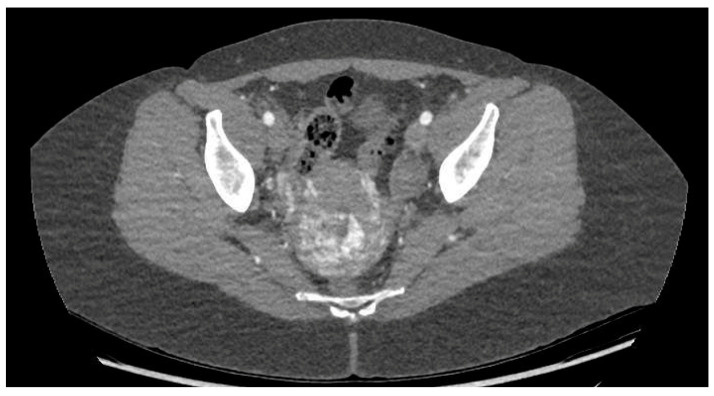
Cross-sectional pelvic CT angiography.

**Figure 4:**
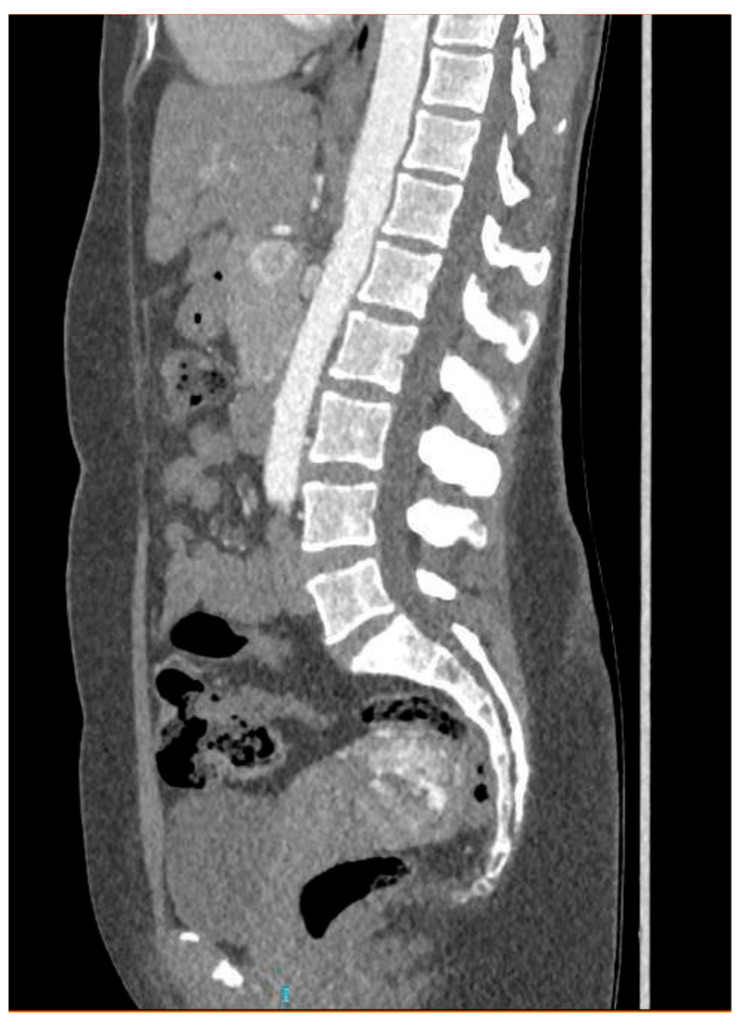
Coronal pelvic CT angiography.

CT angiography showed a complex network of millimetre-sized vessels of tortuous appearance with rapid contrast enhancement in the late arterial phase and isodensity in the portal phase (similar to systemic venous vessels). This network extended through the right parametrium, through the myometrial fundus, and apparently into the endometrium (Figure 3 and Figure 4). The radiological findings were compatible with a uterine arteriovenous malformation.

**Figure 5:**
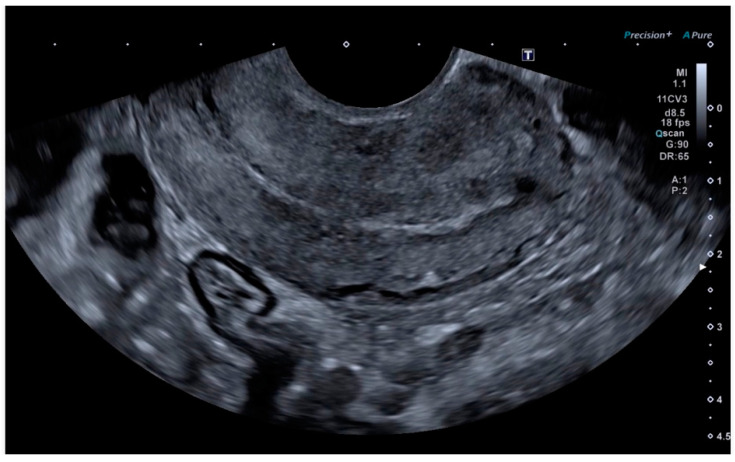
Linear endometrium after conservative treatment for 4 months.

**Figure 6:**
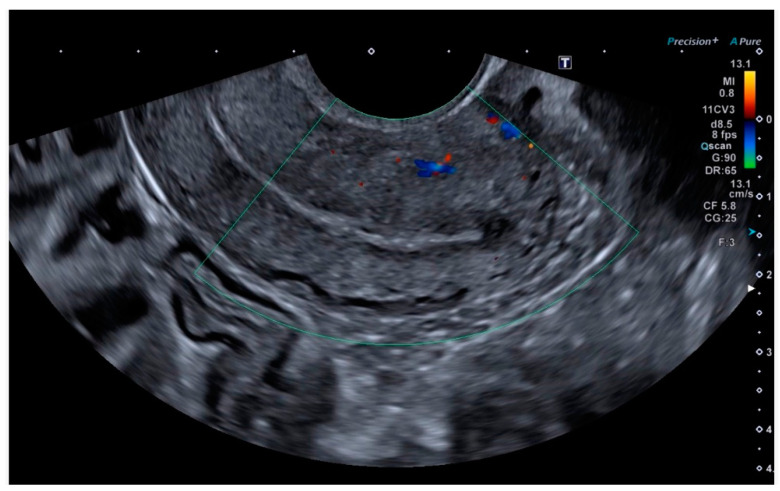
Absence of colour Doppler uptake.

Finally, the vascular surgeons ruled out selective arterial embolisation because the size of the lesion made complete and effective embolisation impossible. Conservative treatment with combined hormonal contraceptives was chosen because the patient’s reproductive desires were not fulfilled. After four months of being asymptomatic and under treatment with combined hormonal contraceptives, the patient presented for follow-up, at which time no AVM was observed on the ultrasound, either in B-mode or colour Doppler (Figure 5 and Figure 6).

## 3. Discussion

The therapeutic approach for this patient was guided by the rapid control of symptoms with conservative treatment and the fact that she had not yet fulfilled her reproductive desires.

AVMs are conditions for which we do not know the prevalence and current incidence in the general population (they are common formations in the pulmonary or cerebral territory but are rarely found in the uterus). Due to the paucity of reported cases and available research, many remain undetected after responding to conservative treatment. They may account for 1–2% of metrorrhagias [8]. It is likely that the frequency of this pathology will increase in the coming years due to the wider use of ultrasound and increased awareness of this pathology.

Histologically, we find fistulae between the myometrial venous plexus and the intramural layer of the arterioles.

Clinically, we can observe this in asymptomatic patients to those with severe metrorrhagia and cardiovascular consequences with the need for haemotransfusions (30%) [9]. In these cases, it is important to make a diagnosis upon suspicion. This will allow us to prolong the study and offer the patient all the therapeutic alternatives available.

### 3.1. Diagnosis of AVMs

Several techniques can be used to diagnose AVMs, such as ultrasound, computed tomography (CT), magnetic resonance imaging (MRI), or angiography, depending on the patient’s condition and the availability of the techniques.

The differential diagnosis of uterine arteriovenous malformations is mainly made by ultrasound: colour and pulsed Doppler [10]. The visualisation of endometrial thickening with an anechoic and vascular intramiometrial structure is very useful in the diagnosis and can help to exclude other causes of dysfunctional uterine bleeding such as retained placental debris, uterine sarcoma, pelvic varices, or gestational trophoblastic disease [11,12,13]. The most common ultrasound findings with colour Doppler are multidirectional myometrial hypervascularisation in the area of the lesion, with turbulent flow and multiple tortuous vessels in the bed [14].

Pulsed Doppler shows low-resistance vessels and high pulsatility indices with a high systolic peak velocity (PSV). In a healthy myometrium, the vessels have a peak systolic velocity of 9–40 cm/s and a resistance index between 0.6 and 0.8, with systolic and diastolic velocities four to six times higher in the case of uterine arteriovenous malformation (PSV between 25 and 110 cm/s with a mean of 60 cm/s and a resistance index of 0.27–0.75 with a mean of 0.41) [15,16].

Depending on the PSV of the lesions, they can be classified as follows [17,18]:

- Mild: PSV < 40 cm/s, where expectant management is recommended.
- Moderate: PSV of 40–60 cm/s, where medical treatment is recommended.
- Severe: PSV > 60–70 cm/s, where arterial embolisation or surgical treatment is recommended.

Therefore, if an AVM is suspected, a Doppler scan should be performed prior to obstetric curettage, as this may worsen the bleeding and even make it fatal, requiring an urgent hysterectomy [1,18].

The use of CT is also widespread but is usually limited to urgent cases due to a patient’s metrorrhagia, when MRI does not provide good resolution, or before surgery to implement the approach plan. One of the advantages of CT over MRI is its better resolution in areas close to bone or the bowels, while the main disadvantage is the radiation it produces.

The images obtained with MRI allow for controlling and monitoring the pathology, including the possibility of assessing arterial supply and venous drainage, as well as high-resolution three-dimensional reconstruction and aiding in therapeutic management.

However, the gold standard for the diagnosis of this pathology is angiography, which is not only a diagnostic but also a therapeutic method. As this technique usually produces high levels of radiation, digital subtraction angiography [19] can be performed with less exposure to ionising radiation and less contrast than conventional studies, with the possibility of obtaining three-dimensional reconstructions of the vessels studied. Nowadays, although angiography is the gold standard, it is reserved for therapeutic embolisation to delay or avoid surgery, while ultrasound is preferred as a diagnostic technique [1,10].

Ultrasound, both two-dimensional B-mode and colour and pulsed Doppler, for the diagnosis of AVMs has proven to be the simplest method with the best diagnostic results [11,20]. It is also the most cost-effective method for patient follow-up [17].

### 3.2. Treatment of AVMs

Treatment options include conservative management with combined hormonal contraception, uterine artery embolisation, or a hysterectomy. An individualised approach to the treatment of AVMs is crucial, as there is no consensus on the superiority of one treatment over another. Treatment is usually selected on the basis of the patient’s symptoms, haemodynamic stability, and the maximum ultrasound PSV of the lesion.

In asymptomatic patients, treatment with norethisterone has been shown to stop bleeding and significantly reduce the lesion in 90% of cases [21]; however, patients with a PSV > 83 cm/s usually require surgical treatment.

In patients who still have reproductive desires and have large AVMs with a PSV > 60–70 cm/s, selective embolisation of the region should be the first choice, provided the patient is haemodynamically stable. This technique has not been shown to affect subsequent pregnancies or placental vascularisation and therefore does not appear to result in intrauterine growth restriction [22,23,24,25]. Embolisation can be repeated if a partial response is achieved, with a success rate of 71–93% [26]. Cases of congenital AVMs have a lower success rate, as they are more extensive lesions that may involve other pelvic and extrapelvic territories.

If embolisation fails, the patient is haemodynamically unstable, or her reproductive wishes are already fulfilled, the last therapeutic option is a hysterectomy. Some cases of fertility preservation surgery, such as uterine artery ligation, have been described without conclusive results [27].

There are numerous cases published in the literature on the surgical management of haemorrhage due to uterine arteriovenous malformations. One published article presented the case of a 32-year-old female patient who arrived to the emergency department 15 days after eutocic delivery with massive haemorrhage. In the immediate puerperium, she presented with severe haemorrhage, requiring the transfusion of two red blood cell concentrates. Transvaginal ultrasound in the emergency department showed a 22 × 44 mm hyperechogenic intrauterine lesion on colour Doppler imaging, compatible with a uterine arteriovenous malformation. Intracavitary tamponade with a Foley catheter was initially used to control the haemorrhage, and it eventually ceased. Conservative management was attempted due to the patient’s age and having only one child. After the vascular surgery team declined to embolise the uterine arteries due to the size of the arteriovenous malformation, a simple total hysterectomy was performed at the patient’s request. [28]

## 4. Conclusions

Uterine arteriovenous malformations are rare, but their incidence is increasing due to both improved diagnosis and the increase in uterine surgery in recent years. The use of ultrasound, both two-dimensional B-mode and colour and pulsed Doppler, is recommended for the diagnosis of AVMs, as it has been shown to be the simplest method with the best diagnostic results. It is also the most cost-effective method for patient follow-up.

As far as treatment is concerned, we need to individualise each case, taking into account haemodynamic stability, the patient’s reproductive wishes, and the severity of the AVM, as assessed by its size and SPV. This assessment will lead us to opt for conservative treatment in mild cases where the patient wishes to conceive, whereas in cases where the patient’s life is at risk, there is no reproductive desire, or the size of the lesion prevents pregnancy, we will opt for embolisation or surgical treatment.

## Data Availability

Data is available within the article.
